# Genome-Wide Association Study of Peripheral Arterial Disease in a Japanese Population

**DOI:** 10.1371/journal.pone.0139262

**Published:** 2015-10-21

**Authors:** Mitsuru Matsukura, Kouichi Ozaki, Atsushi Takahashi, Yoshihiro Onouchi, Takashi Morizono, Hiroyoshi Komai, Hiroshi Shigematsu, Toshifumi Kudo, Yoshinori Inoue, Hideo Kimura, Akihiro Hosaka, Kunihiro Shigematsu, Teturo Miyata, Toshiaki Watanabe, Tatsuhiko Tsunoda, Michiaki Kubo, Toshihiro Tanaka

**Affiliations:** 1 Laboratory for Cardiovascular Diseases, RIKEN Center for Integrative Medical Sciences, Yokohama, Japan; 2 Division of Vascular Surgery, Department of Surgery, Graduate School of Medicine, The University of Tokyo, Tokyo, Japan; 3 Laboratory for Statistical Analysis, RIKEN Center for Integrative Medical Sciences, Yokohama, Japan; 4 Laboratory for Medical Science Mathematics, RIKEN Center for Integrative Medical Sciences, Yokohama, Japan; 5 Unit of Peripheral Vascular Surgery, Department of Surgery, Kansai Medical University, Osaka, Japan; 6 Clinical Research Center for Medicine, International University of Health and Welfare, Tokyo, Japan; 7 Department of Professional Development, Tokyo Medical and Dental University, Tokyo, Japan; 8 Department of Vascular Surgery, Department of Esophageal and General Surgery, Tokyo Medical and Dental University, Tokyo, Japan; 9 Department of Surgery, Manaka-hospital, Kanagawa, Japan; 10 Department of Surgery, Tokyo Metropolitan Tama Medical Center, Tokyo, Japan; 11 Sanno Hospital and Sanno Medical Center, Vascular Center, Tokyo, Japan; 12 Department of Surgical Oncology, Graduate School of Medicine, The University of Tokyo, Tokyo, Japan; 13 Laboratory for Genotyping Development, RIKEN Center for Integrative Medical Sciences, Yokohama, Japan; 14 Bioresource Research Center, Tokyo Medical and Dental University, Tokyo, Japan; National Institute of Environmental Health Sciences, UNITED STATES

## Abstract

Characteristics of peripheral arterial disease (PAD) are the occlusion or stenosis of multiple vessel sites caused mainly by atherosclerosis and chronic lower limb ischemia. To identify PAD susceptible loci, we conducted a genome-wide association study (GWAS) with 785 cases and 3,383 controls in a Japanese population using 431,666 single nucleotide polymorphisms (SNP). After staged analyses including a total of 3,164 cases and 20,134 controls, we identified 3 novel PAD susceptibility loci at *IPO5/RAP2A*, *EDNRA* and *HDAC9* with genome wide significance (combined *P* = 6.8 x 10^−14^, 5.3 x 10^−9^ and 8.8 x 10^−8^, respectively). Fine-mapping at the *IPO5/RAP2A* locus revealed that rs9584669 conferred risk of PAD. Luciferase assay showed that the risk allele at this locus reduced expression levels of *IPO5*. To our knowledge, these are the first genetic risk factors for PAD.

## Introduction

Peripheral artery disease (PAD) is characterized by the obstruction of the blood supply to multiple sites including carotid, mesenteric, renal, upper and lower extremities mainly caused by atherosclerosis [1]. Chronic ischemic change of lower extremity arteries are the most common condition of the disease and cause serious impairment and reduced quality of life. People with PAD are also known to have roughly a three-fold increase in risk of major cardiovascular events and mortality compared with those without PAD [2–6]. PAD is now estimated to be the third leading cause of death of atherosclerotic-related vascular disease. The number of PAD patients has increased by more than 20% over the past decade, and its prevalence is expected to increase worldwide [7]. Previously known risk factors for PAD include gender, age and smoking, and is also associated with conditions such as hypertension, dyslipidemia, and diabetes mellitus [8–10]. Although these conditions themselves have a genetic susceptibility component, positive PAD family history has been shown to be an independent predictor of the disease [11–14]. The Swedish twin registry reported a high risk of disease among those whose twin had PAD and estimated the genetic effect and non-shared environmental effect to account for 58% and 42% of the variation in incidence, respectively [11]. In addition to sibling studies, a number of candidate gene and linkage analysis studies have been performed in PAD, but are collectively still inconclusive [15]. Since PAD is considered a polygenic disease influenced by multiple environmental factors, a more systematic approach is required to identify genetic factors. Here, we report three loci associated with PAD susceptibility based on a GWAS conducted in a Japanese population.

## Materials and Methods

### Study populations

The majority of case and control samples included in this GWAS and follow-up stages were obtained from BioBank Japan [16]. A subset of the replication case samples were obtained from the Tokyo Medical University Hospital, The University of Tokyo Hospital and affiliated hospitals from Sep 2009 to Sep 2014. Characteristics of the study subjects were summarized in Table 1.

**Table 1:** Characteristics of Study Subjects.

Sample	Subjects	Age ± SD	Male %	ABI ± SD	BMI ± SD	Smoking %	HT %	CAD%	DM%	HL%
**GWAS**	PAD	70.4 ± 9.5	79.8	0.69 ± 0.20	22.6 ± 3.3	81.1	70.7	37.9	46.7	37.9
	Controls	51.6 ± 16.6	55.4	-	22.5 ± 3.7	54.0	28.3	3.3	8.1	0
**Replication**	PAD	70.6 ± 9.1	77.8	0.73 ± 0.22	22.7 ± 3.5	78.9	80.9	45.0	38.6	34.9
	Controls	61.1 ± 12.8	43.9	-	22.2 ± 3.4	47.3	26.2	0	0	0

ABI; ankle-brachial index, BMI; body mass index, HT; hypertension, CAD; coronary artery diseases, DM; diabetes mellitus, HL; hyperlipidemia

The BioBank Japan project (see URLs) commenced in 2003 for the collection of genomic DNA, serum and clinical information from approximately 300,000 cases diagnosed with any of 47 diseases by a collaborative network of 66 hospitals in all areas of Japan. We used PAD cases that were collected from May 2003 to December 2006 in BioBank Japan for GWAS analysis after checking the clinical information. All the cases were diagnosed as PAD on the basis of the clinical information. We included PAD patients with ABI index < 0.9 used or a Fontaine class of IIa more or history of PAD therapy (stent, atherectomy and other surgical treatment). All study subjects provided written informed consent to participate in this study. The consent was obtained for the banking when we enrolled. The protocol was approved by the RIKEN Yokohama Campus Ethics Committee, Research Ethics Committee/Human Genome, Gene Analysis Research Ethics Committee of Graduate School of Medicine of the University of Tokyo, and the University of Tokyo hospital, Tokyo Medical University's Ethics Committee, Ethics committee of Kansai Medical University, Ethics committee of Kyorin University, Ethics committee of Ome Municipal General Hospital and Human Genome, Gene Analysis Research Ethics Committee of Ibaraki prefectural Central Hospital.

### SNP genotyping

Samples were genotyped by the Illumina HumanHap610-Quad BeadChip for the cases and Illumina HumanHap550v3 for controls. We applied stringent quality-control criteria and tested 785 cases and 3,383 controls for 497,509 autosomal SNPs commonly available on both BeadChips. In the GWAS, we applied SNP quality control (call rate of ≥0.99 in both cases and controls and Hardy-Weinberg equilibrium test *P* ≥ 1.0 × 10^−6^ in controls); 431,666 SNPs on all chromosomes passed the quality control filters and were further analyzed. All control samples for the follow-up stage were genotyped using the Illumina HumanHap610-Quad BeadChip. To confirm the accuracy of Illumine genotyping at rs9584669, we conducted direct genotyping using Invader assay (Third Wave Technologies) [17] and found no inconsistency between the results of Invader assay and Illumina genotyping. All cluster plots were checked by visual inspection by trained personnel, and SNPs with ambiguous calls were excluded. For cases in the follow-up stage, we used the multiplex PCR-based Invader Assay.

### Statistical analyses

The analysis of the association between SNPs and PAD were assessed with the Cochran-Armitage trend test. Significance level of the GWAS was set at 1.2 x 10^−7^ (0.05/431,666) after Bonferroni correction for multiple testing. To further validate the results of the GWAS, we selected the 500 SNPs with the most significant Cochrane-Armitage trend p values for replication analyses in additional 1,150 cases and 16,752 controls. Of the selected 500 SNPs, 145 showed evidence of strong linkage disequilibrium (*r* ^2^ > 0.8) with other selected markers as assessed by the Haploview software. We thus selected 355 SNPs for further genotyping. Combined analysis was performed using the Mantel-Haenszel method. We examined the inflation of test statistics, λ_genomic control_ (λ_gc_) by genomic control method [18]. We also conducted principal component analysis (PCA) to assess population stratification using the GWAS data [19]. We obtained the other genotype data from the Phase II HapMap database. Relationships between clinical profiles and genotype of the cases were examined by χ^2^ test for gender difference and coronary risk factors, and one-way ANOVA for quantitative clinical parameters.

### Fine-mapping

We carried out Sanger sequencing for a 100kb region around rs9584669 (chromosome position (NCBI build 38); 97,658,003–97,758,002) using 48 case samples. We found a total of 249 SNPs and conducted re-sequence using additional 48 case samples. We selected 191 SNPs of MAF ≥ 0.05 and chose 25 tag SNPs. We also analyzed the LD pattern and determined the LD block using the Haploview software. We conducted invader assay for all tag SNPs using GWAS case samples (n = 750) and a subset of GWAS control samples (n = 2,418). Statistical analysis was performed using the Mantel-Haenszel method.

### Cells

Human aortic smooth muscle cells (HASMC, Gibco^®^ Invitrogen cell culture) were cultured in Smooth Muscle Cell Medium (SMCM, ScienCell research laboratories) and maintained at 37°C in atmospheres of humidified air with 5%CO_2_.

### Luciferase assay

We checked the H3K27Ac sequences of the *IPO5/RAP2A* region on chromosome 13q32.2 (UCSC genome browser; http://genome.ucsc.edu) and cloned genomic fragments for the H3K27Ac sequences (chromosome position (NCBI build 38); 97,980,373–97,980,941 for *IPO5* and 97,428,261–97,428,846 for *RAP2A*) to the multiple cloning site of the pGL3 basic vectors (Promega). We confirmed a marked increase of luciferase activity compared to the empty pGL3 basic vector. Then we prepared 25 base pair double stranded oligonucleotides (S1 Table) including the target SNPs of interest (rs9584669, rs9556806, rs9805548, rs9556797, rs9556705, rs4001162, rs9556799) and inserted each of them to the *IPO5/RAP2A* H3K27Ac sequence cloned pGL3 vectors. We transfected these constructs in human aortic smooth muscle cells (HASMC) using the nucleofector^TM^ system (Amaxa). Forty-eight hours after transfection, we analyzed the luciferase activity using the dual- luciferase reporter assay system according to the manufacturer’s protocol. (Promega Corporation, Wisconsin, USA) and luminomater (Centro LB960, BERTHOLD TECHNOLOGIES GmbH & Co. KG). The relative *firefly*/*Renilla* luciferase value was calculated for each sample and standardized each value based on the value of the *IPO5/RAP2A* H3K27Ac sequence cloned pGL3 vectors in the same experiment. The empty pGL3-basic vector was used as a negative control. Each experiment was independently performed three times and each sample was studied in duplicate. Student’s t-test was conducted to estimate statistical difference of non-risk allele and risk allele activity.

### Software

For general statistical analysis, we used R statistical environment version 2.10.0. or PLINK1.05 [20]. To draw the LD map, we used Haploview software [21]. To make regional maps, we used Locus zoom software.

### URLs

BioBank Japan project; http://biobankjp.org/. HapMap project, http://hapmap.ncbi.nlm.nih.gov/. PLINK 1.05, http://pngu.mgh.harvard.edu/~purcell/plink/. R software, http://www.r-project.org/; LocusZoom, http://csg.sph.umich.edu/locuszoom/; eQTL database, http://www.hsph.harvard.edu/liming-liang/software/eqtl/.

## Results

### GWAS

**Fig 1:**
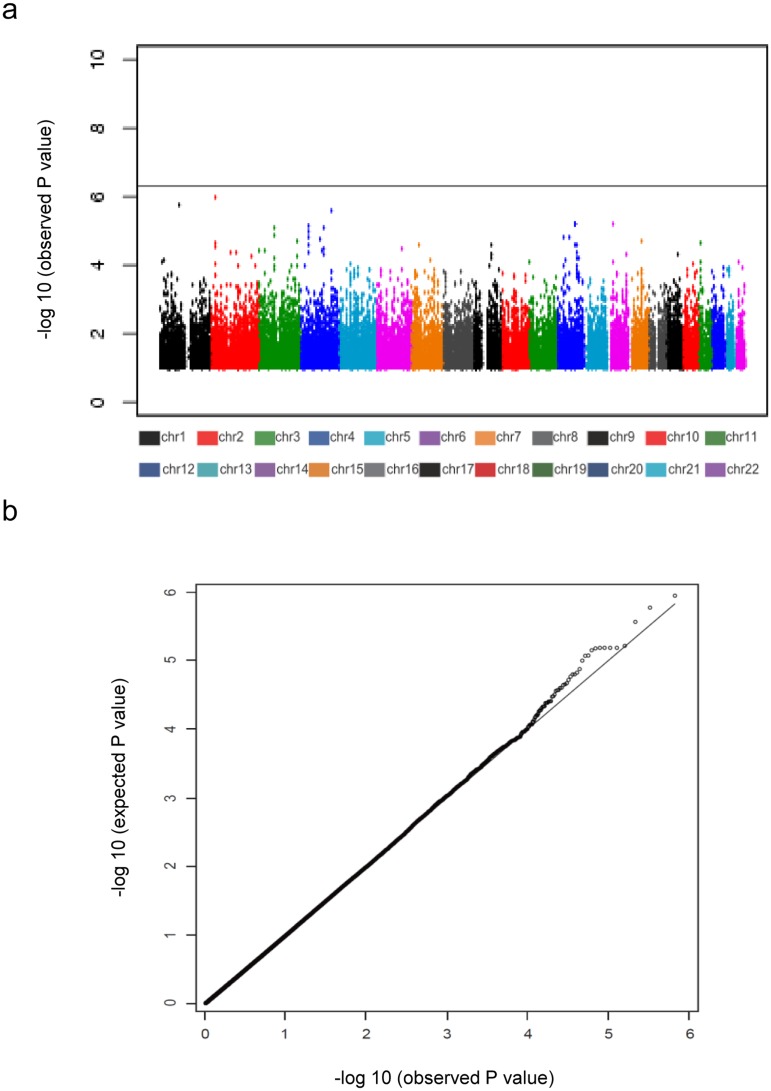
Manhattan plot (a) and quantile-quantile plot (b) of the GWAS.

**Fig 2:**
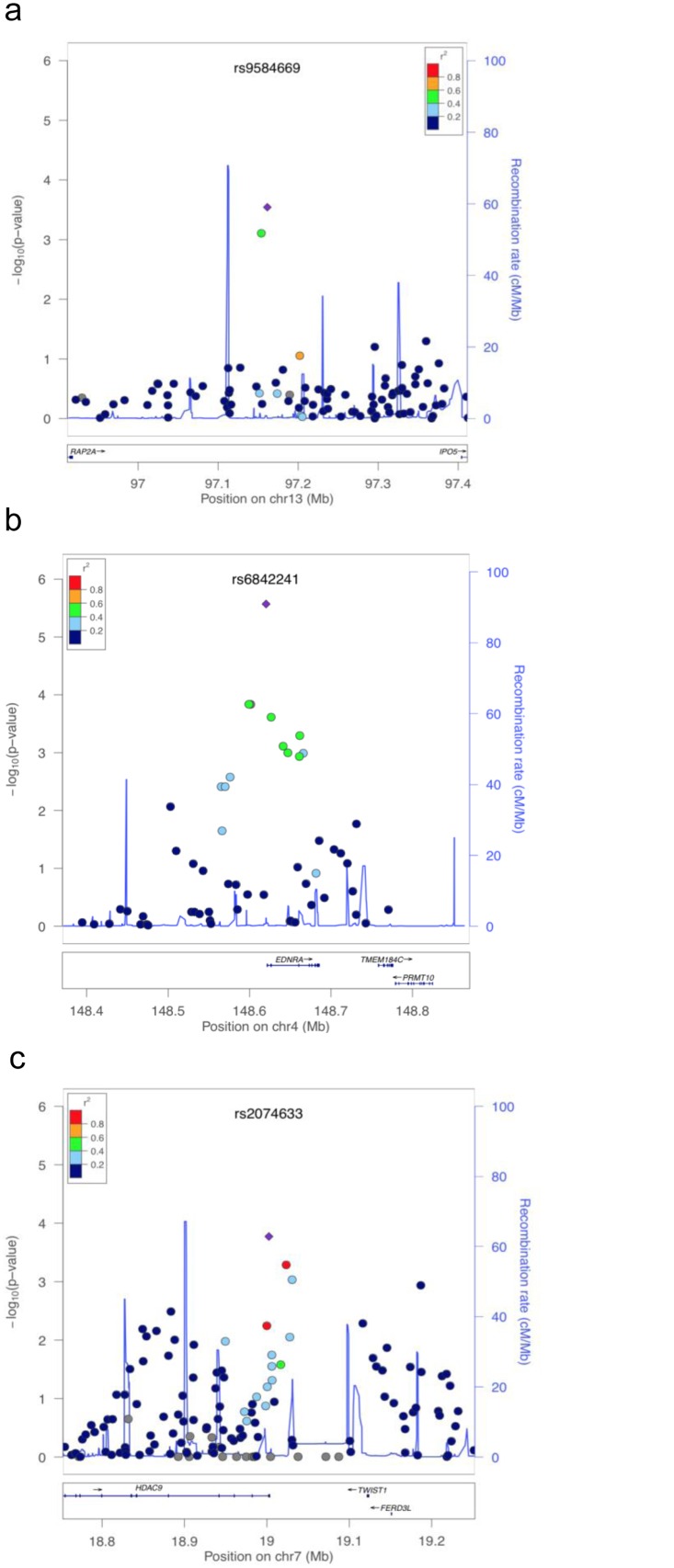
Regional plots of susceptible loci on 13q32.2 (a), 4q31.2 (b), and 7p21.1 (c). Estimated LD structure of the genomic region in the JPT HapMap samples is shown as light-blue lines, and the genomic locations of genes within the regions of interest were annotated using the UCSC Genome Browser and are shown as arrows. SNPs are colored according to their LD with the tag SNP. Diamonds in red represent the most significantly associated SNP in each region in the GWAS.

To identify novel PAD susceptible loci, we performed a GWAS for PAD with a Japanese population consisting of 785 cases and 3,383 controls. We evaluated the presence of population stratification by comparison to HapMap samples using principal component analyses and found that all cases and controls clustered among the Asian population and almost all subjects fell into the two main known clusters of the Japanese general population (S1 Fig). We examined the association between SNP genotypes and PAD using the Cochran-Armitage trend test (S2 Table). Fig 1a indicated-log_10_ *P* values of the 431,666 SNPs we examined. In this GWAS, no SNP reached the threshold for statistical significance based on a Bonferroni correction (*P* < 1.2 x 10^−7^). The inflation of test statistics, λ_genomic control_ (λ_gc_) was 1.04 (Fig 1b). To further explore the suggestive loci, we decided to focus on the top 500 SNPs ranked by p-value in the GWAS which was reduced to 355 loci after considering linkage disequilibrium (LD). We then genotyped another panel of 1,150 cases and 16,752 controls, and 13 SNPs showed a p < 0.0001 (S3 Table). For these loci, an additional 1,229 cases were examined, expanding the total number of PAD cases to 3,164 (S4 Table). By combination of P values for these association analyses, we identified three loci that were associated with PAD, in close proximity to the genes *IPO5/RAP2A*, *EDNRA* and *HDAC9* (Table 2, Fig 2).

**Table 2:** Summary of Association with the Risk of PAD.

dbSNP ID	Chr.	Gene	Phase	Number of samples		MAF		OR	95%CI	*P*
				Cases	Controls	Cases	Controls			
**rs9584669**	13	*IPO5*/RAP2A	GWAS	785	3383	0.04	0.06	0.60	0.45–0.79	2.76 x 10^−4^
			Replication	2379	16751	0.03	0.05	0.57	0.48–0.66	2.10 x 10^−11^
			Combined*	3164	20134	0.03	0.05	0.58	0.50–0.66	6.78 x 10^−14^
**rs6842241**	4	*EDNRA*	GWAS	785	3372	0.36	0.30	0.76	0.68–0.85	2.36 x 10^−6^
			Replication	2342	16750	0.33	0.30	0.88	0.83–0.93	9.09 x 10^−5^
			Combined*	3127	20122	0.34	0.30	0.85	0.80–0.90	5.32 x 10^−9^
**rs2074633**	7	*HDAC9*	GWAS	785	3382	0.43	0.38	1.24	1.11–1.38	1.41 x 10^−4^
			Replication	2363	16751	0.41	0.38	1.13	1.06–1.20	7.63 x 10^−5^
			Combined*	3148	20133	0.41	0.38	1.16	1.10–1.22	8.43x 10^−8^

ID; identifier, Chr.; chromosome, MAF; minor allele frequency, OR; odds ratio, CI; confidence interval.

*; P value was calculated by Mantel-Haenszel test.

We explored the possibility of confounding effects by age, gender, and classical risk factors including diabetes, hypertension, smoking, and hyperlipidemia within the patient group using one-way ANOVA and x^2^ test for three significant SNPs, and found no obvious relation between genotypes and these factors. This indicated that these three SNP loci were independent of these lifestyle for PAD in the Japanese population (S5 Table).

### Fine mapping of 13q32 locus

As the identified SNPs on the top locus are located within the flanking region of two genes, we further investigated which gene might relate to the disease susceptibility through fine mapping followed by *in vitro* functional analysis. To narrow down the *IPO5/RAP2A* locus on chromosome 13q32.2, we performed direct sequencing of the region with 48 case samples, and identified 249 SNPs within a 100kb LD region. Among them, we selected 24 tag SNPs that represent this locus for further fine-mapping. Association analysis of these tag SNPs with 750 cases and 2405 controls revealed rs9584669 to have the strongest p value (Cochran-Armitage trend test) (S6 Table). Genotyping also revealed that six SNPs with the 13q32.2 genomic region (rs9556806, rs9805548, rs9556797, rs9556705, rs4001162, rs9556799) were in absolute LD with this SNP (rs9584669).

### Functional analysis of the associated SNP on chr.13q32

**Fig 3:**
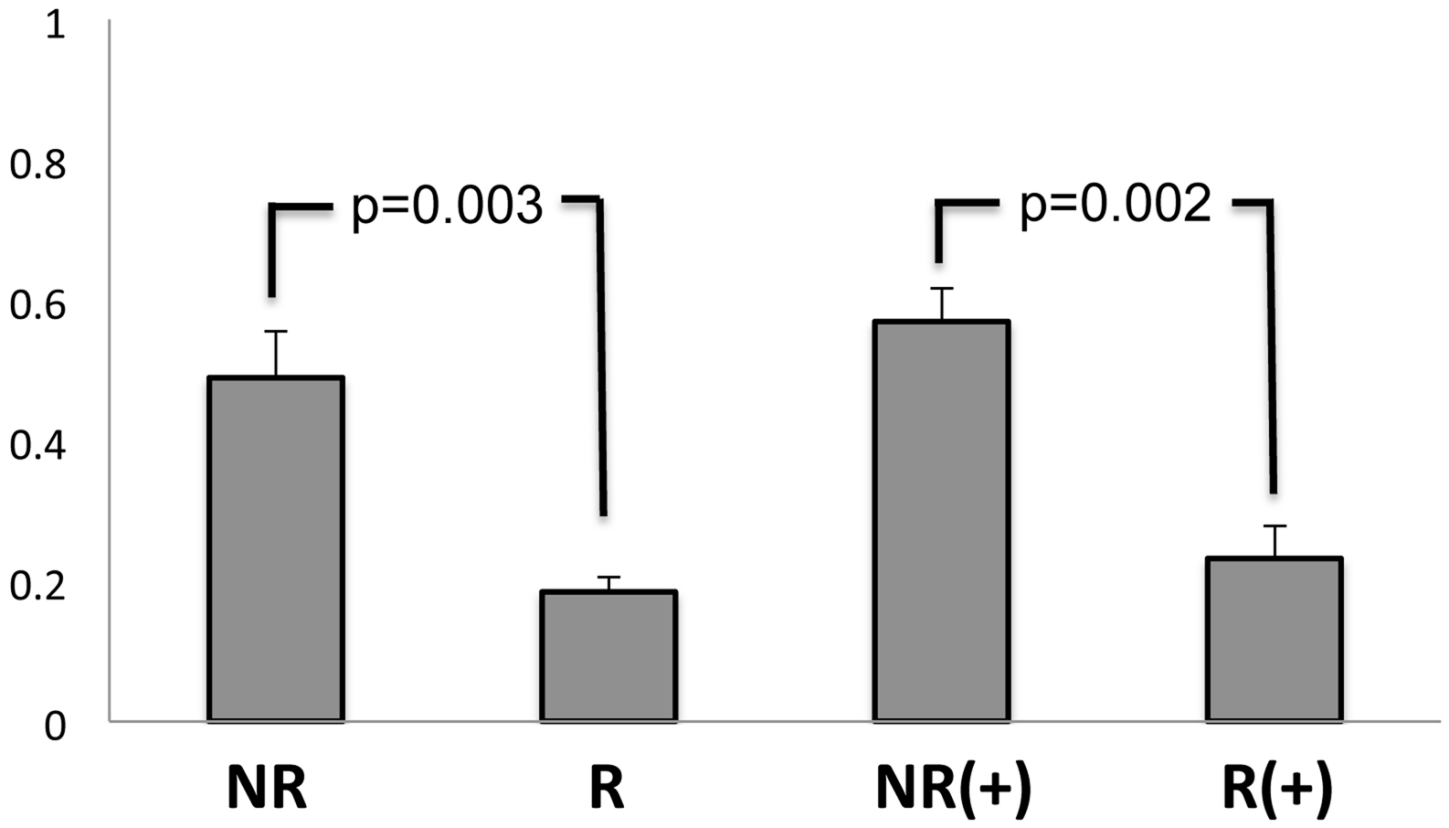
Luciferase assay for rs9584669-*IPO5* promotor constructs. A clone containing the rs9584669 SNP non-risk allele had an approximately 1.5-fold greater transcriptional activity than those containing the risk allele in H3K27Ac mark for *IPO5* and was evaluated using the student's t-test. NR and R indicate non-risk and risk alleles, respectively. (+) shows the result after six hours of stimulation by Ionomycin and PMA.

Since none of these 7 SNPs within this associated region account for a change in amino acid sequence of the protein, we investigated whether these SNPs would affect *IPO5* and/or *RAP2A* expression using a reporter gene analysis in human aortic smooth muscle cells (HASMC). We used this cell because abundant expression of both *IPO5* and *RAP2A* mRNA were observed in quantitative RT-PCR experiments. Fig 3 showed that only clones containing the rs9584669 SNP non-risk allele had an approximately 1.5-fold greater transcriptional activity than those containing the risk allele in the *IPO5* promoter construct. From this result, we hypothesized that the rs9584669 SNP genomic locus physically interacts to the promoter region of *IPO5* in a long range looping manner, and the transcriptional repressive factor(s) which interacts strongly with the genomic complex includes the risk SNP suppress transcription of *IPO5*. No allelic difference was observed in other *IPO5* promoter constructs and the *RAP2A* promoter constructs (S3 Fig). These results indicate that the associated SNP affected the transcription level of *IPO5*, but not *RAP2A*.

## Discussion

Importin-5 (IPO5) is a member of the importin beta family [22] and is mainly localized in the cytoplasm, especially in the nuclear pore complex. This protein transports nuclear localization signal (NLS)-containing cargo from cytoplasm to nucleus in the presence of nucleoside triphosphates and the small GTP binding protein Ran [23]. One important role of IPO5 is to promote excretion of apolipoprotein A-1 [24], a major protein component of HDL particles and mediates transport of lipids from peripheral tissues as part of the reverse cholesterol transport pathway. Apolipoprotein A-1 also controls off-load of cholesterol esters from HDL particles to liver through scavenger receptor B1 and to LDL via cholesterol ester transfer protein. Through these processes, HDL reduces the accumulation of plaque inside of blood vessel intima [25]. Since a clinical feature of PAD involves atherosclerotic change of the mid to small arteries, potential of HDL function may be key to preventing disease progression. In this sense, rs9584669 allele may reflect anti-atherosclerotic ability of each individual.

Another significant SNP, rs68422241, was located in the 5’ flanking region of the endothelin receptor type A gene (*EDNRA*). *EDNRA* encodes a receptor for endothelin-1 [26], a peptide that plays a role in potent and long-lasting vasoconstriction [27] and pro-inflammatory effects [28]. Endothelin-1 mediates activation of vascular smooth muscle cells (VSMC) and showed increased expression in human atherosclerotic lesions [29, 30], indicating that endothelin-1 contributes to the pathogenesis of chronic inflammation associated with atherosclerosis. Endothelin-1 also induces the release of inflammatory cytokines, including interleukin (IL)-6, IL-1β and C-reactive protein, from VSMC and/or monocytes [31–33]. Signaling of a receptor for endothelin-1, EDNRA, mediates activation and proliferation of VSMC, and its selective inhibitors prevent endothelial dysfunction, structural vascular change in atherosclerosis, and also inhibit cholesterol induced atherosclerosis [34–36]. Furthermore, other studies indicated that EDNRA antagonists also reduced the adhesion of inflammatory cells to endothelial cells through inhibition of adhesion molecule expression [37, 38]. Together with our genetic evidence, these known findings also suggest that the endothelin-1—EDNRA cascade has an important role in the development and progression of PAD. It is of note that a functional genetic variant of *EDNRA* was also associated with other vascular diseases including coronary artery disease [39], intra cranial aneurysm [40] and ischemic stroke [41].

We also found an association with a SNP, rs2074633, near the *HDAC9* gene encoding histone deacetylase-9. Histone deacetylase (HDAC) is a component of multiprotein complexes and modulate transcription events; thus, the HDAC family plays a critical role in transcriptional regulation and cell cycle progression [42]. HDAC9 classifies as a class IIa enzyme and is known to shuttle between the nucleus and the cytoplasm [43]. Another function of this protein is related to tissue remodeling via the stress-response pathway [44–46]. A genetic variant in *HDAC9* is also associated with large vessel ischemic stroke, carotid atherosclerosis and coronary artery disease [41, 47, 48]. Since severe intimal thickness of mid to small arteries are characteristics of PAD, combined with these previous findings, it is possible that HDAC9 may be involved in the pathogenesis of this disorder and its progression.

Through a GWAS and a subsequent replication study in Japanese subjects, we identified three novel PAD susceptible loci near the genes *IPO5/RAP2A*, *EDNRA* and *HDAC9*. Because of the genetic difference among ethnicity, the relevance of our findings to other ethnic groups remains to be clarified. Another limitation might be that, we might miss some positive results because of the relatively small sample size at initial GWAS stage. To our knowledge, our study is the first to identify these loci for PAD through GWAS. We believe that knowledge of genetic risk factors and their molecular cascade associated with PAD will contribute to a better understanding of the pathogenesis and aid in early detection of PAD.

## Supporting Information

S1 Fig **(a)** Principal components analysis (PCA) of population in the GWAS. The relatedness among cases and controls for GWAS along with the European (CEU), African (YRI), and East-Asian (JPT and CHB) data from the HapMap project was analyzed. The individuals were plotted in a two-dimensional graph,with the first (x axis) and the second (y axis) components of the Eigenvector factors. **(b)** The relatedness, along with the East-Asian (JPT and CHB) data from the HapMap project was analyzed.
(PDF)

S2 Fig LD structure at chr. 13q32.2 locus Linkage disequilibrium plot for rs9584669 including all SNPs within 100kb region. Tag SNPs are circled.
(PDF)

S3 Fig Result of Dual reporter Luciferase assay for *IPO5* (a) and *RAP2A* (b). No allelic diffrence of transcriptional activity was observed in all the 13SNPs.
(PDF)

S1 Table Oligo nucleotide sequences used for luciferase assay. (XLSX)

S2 Table Results of GWAS. OR; odds ratio, *P* values were caluculated Cochran-Armitage trend test.
(XLSX)

S3 Table Association results of 355 loci ranked by p-value in the GWAS. OR; odds ratio, NG; no good; Combined *P* value was calculated Mantel-haenszel test.
(XLSX)

S4 Table Association results of the 13 SNPs. ID; identifier, Chr; chromosome, OR; odds ratio, CI; confidence interval; Combined *P* value was calculated by Mantel-Haenszel test.
(XLSX)

S5 Table Association between conventional risk factors for PAD and 3 SNPs in cases. HT, hypertension; DM, diabetes mellitus; HL, hyperlipidemia; BI, brain infarction; CAD, coronary artery disease, * For age, mean ± SD is shown.
(XLSX)

S6 Table Association study of tag SNPs at chromosome 13q32 region. ID; identifier, MAF; minor allele frequency, OR; odds ratio, *; *P* value was calculated by Cochran-Armitage trend test.
(XLSX)
