# Supplementary figures and images for: Genome-Wide Association Study of Peripheral Arterial Disease in a Japanese Population
Source: PLoS One. 2015 Oct 21;10(10):e0139262. doi: 10.1371/journal.pone.0139262 (PMC4619060; doi:10.1371/journal.pone.0139262)

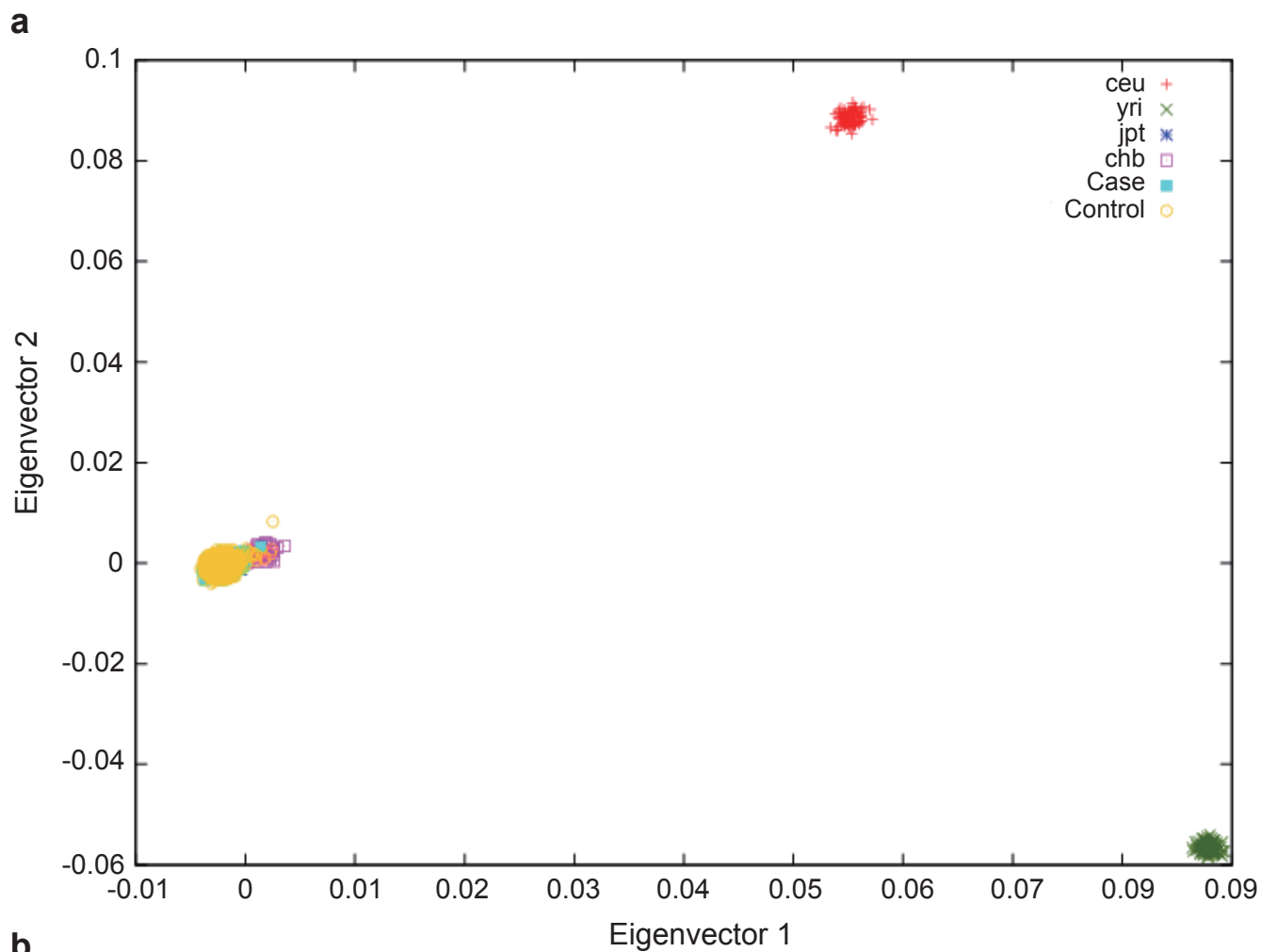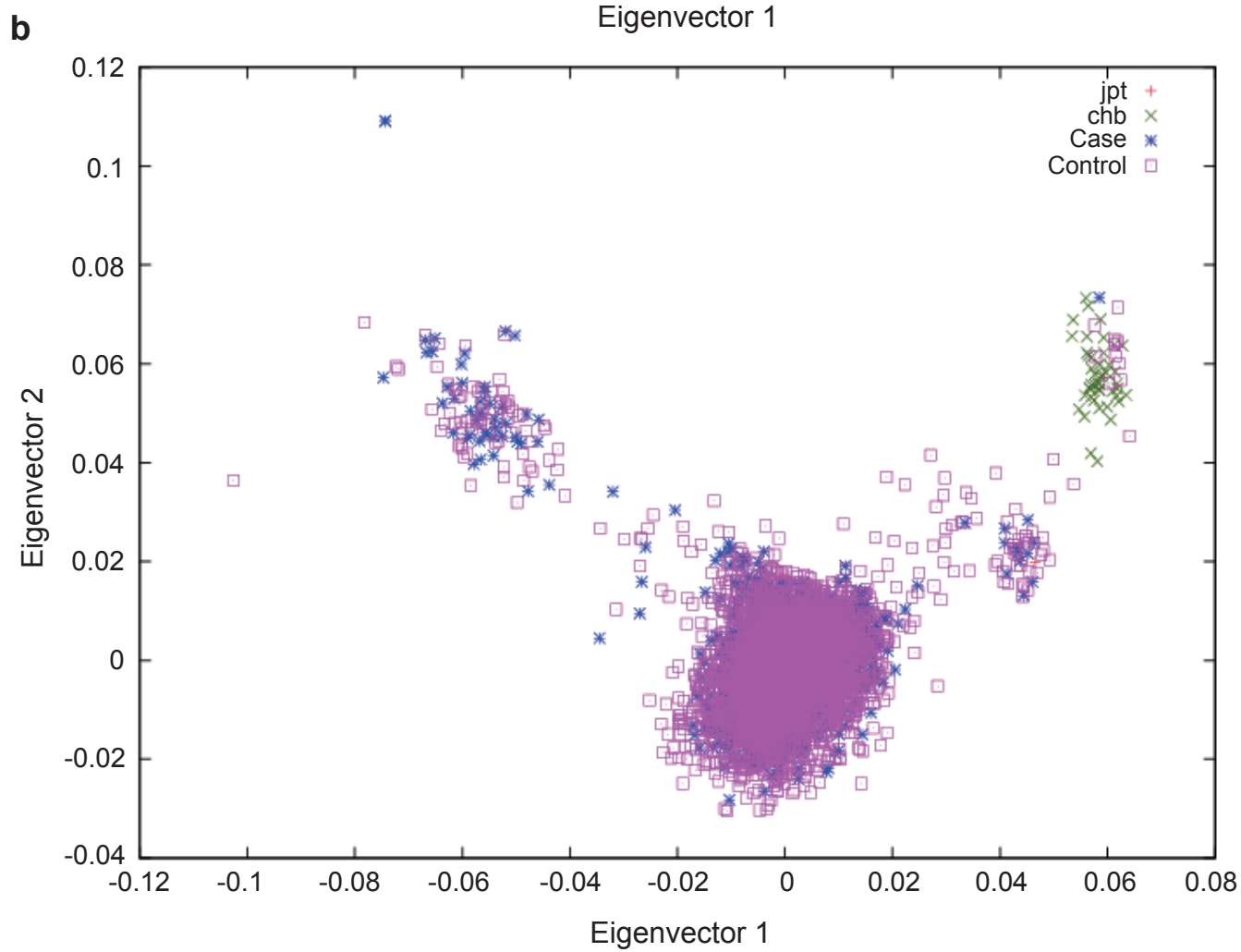

Supplement: S1 Fig — (a) Principal components analysis (PCA) of population in the GWAS. The relatedness among cases and controls for GWAS along with the European (CEU), African (YRI), and East-Asian (JPT and CHB) data from the HapMap project was analyzed. The individuals were plotted in a two-dimensional graph,with the first (x axis) and the second (y axis) components of the Eigenvector factors. (b) The relatedness, along with the East-Asian (JPT and CHB) data from the HapMap project was analyzed. (PDF) [file pone.0139262.s001.pdf]

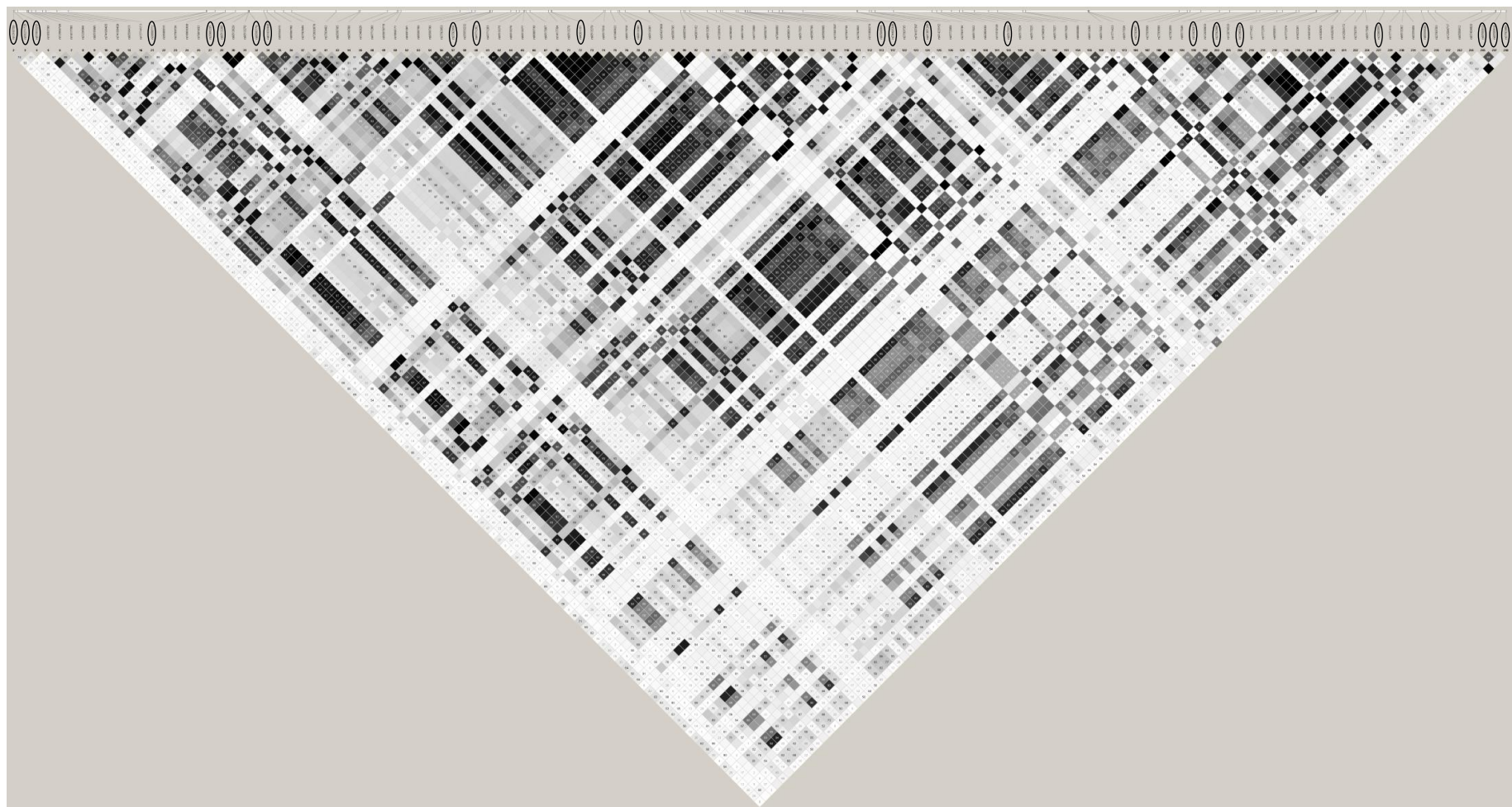

Supplement: S2 Fig — Linkage disequilibrium plot for rs9584669 including all SNPs within 100kb region. Tag SNPs are circled. (PDF) [file pone.0139262.s002.pdf]

**a**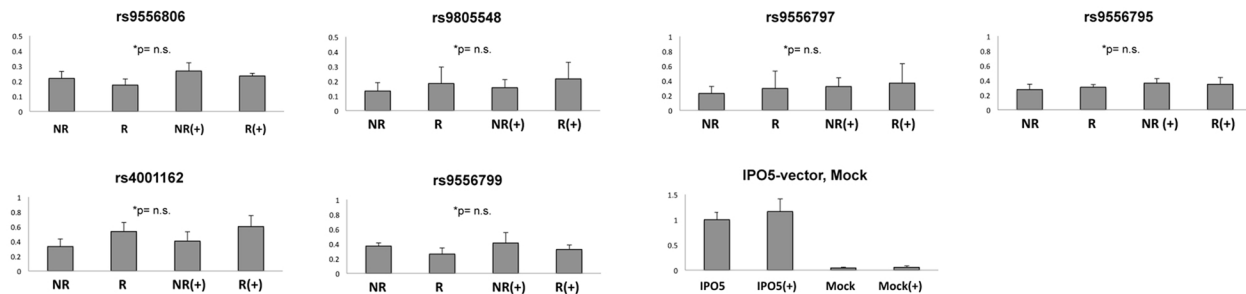**b**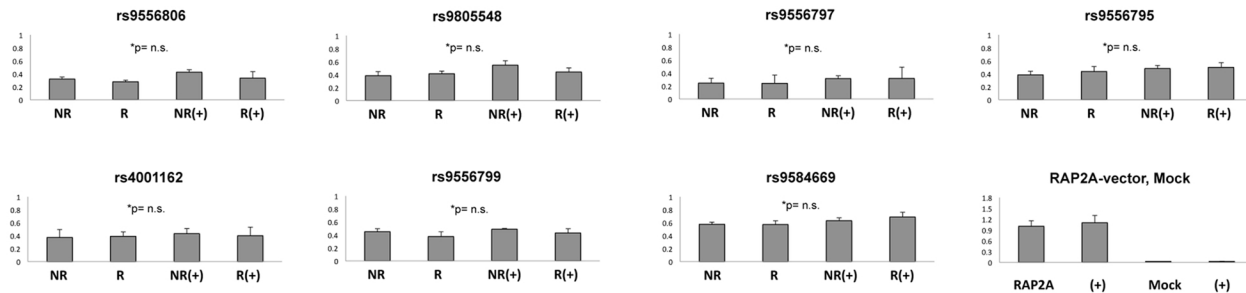

Supplement: S3 Fig — No allelic diffrence of transcriptional activity was observed in all the 13SNPs. (PDF) [file pone.0139262.s003.pdf]
